# DiAbot: A Conversational AI System Coupling Large Language Models with an Interpretable Decision Tree for CDR-Style Dementia Screening

**DOI:** 10.3390/bioengineering13091013

**Published:** 2026-08-31

**Authors:** Hala Alshamlan

**Affiliations:** Department of Information Technology, College of Computer and Information Sciences, King Saud University, P.O. Box 51178, Riyadh 11543, Saudi Arabia; halshamlan@ksu.edu.sa

**Keywords:** Clinical Dementia Rating, CDR, Alzheimer’s disease, decision tree, explainable AI, ADNI, conversational agent, large language model, dementia staging, bioengineering

## Abstract

Alzheimer’s disease and related dementias are projected to affect more than 150 million people worldwide by 2050. Early staging with validated instruments such as the Clinical Dementia Rating (CDR) scale is essential for timely intervention, yet access to clinician-administered CDR assessment remains constrained by workforce, time, and geographic barriers. This study complements a previously published machine learning pipeline for Alzheimer’s disease prediction by addressing the downstream task of dementia staging. Because the global CDR score is already derived from the six sub-domain ratings through an established rule-based procedure, the contribution reported here lies not in discovering that mapping but in encoding it in a transparent, deployable form: an explainable decision tree classifier embedded in DiAbot, a large-language-model-fronted conversational system that supports self-administered CDR-style assessment. We extracted 13,453 CDR records from the Alzheimer’s Disease Neuroimaging Initiative (ADNI), removed administrative variables, invalid entries, and missing rows (final n = 13,290), and trained decision tree classifiers under two impurity criteria, Information Gain and Gini Index, using a 70/30 stratified record-level hold-out and ten-fold stratified record-level cross-validation. This classifier-level evaluation uses the six domain scores as recorded during ADNI’s clinician-administered assessment, not scores elicited by the DiAbot chatbot; the trained classifier was separately embedded in a web application in which a prompt-engineered large language model conducts a CDR-style interview and normalizes responses to ordinal domain scores, but the end-to-end accuracy of that full conversational pipeline (chatbot elicitation through to final CDGLOBAL) has not yet been measured, and is not what the headline accuracy figures below report. The Information Gain Decision Tree reproduced the established mapping from the six CDR sub-domain scores to the CDGLOBAL with 99.86% accuracy under the record-level hold-out protocol (matching macro-averaged precision, recall, and F1-score), with a ten-fold record-level cross-validated mean of 99.81% (SD 0.07); this result represents fidelity to the established CDR scoring rule rather than independent dementia-diagnosis accuracy. Gini-based trees performed almost identically (99.79% hold-out, 99.74% cross-validated). Memory dominated feature importance, consistent with its role as the primary domain in the official CDR scoring algorithm. Residual misclassifications were confined to adjacent CDR stages. Because the CDGLOBAL is deterministically derived from the six sub-domain scores, these figures should be read throughout as evidence of high-fidelity reconstruction of the established CDR scoring relationship, not as general dementia-diagnosis accuracy comparable to imaging- or biomarker-based classifiers; further, participant-independent generalization remains unverified under the record-level protocol evaluated here. An interpretable classifier embedded in a conversational front-end can nonetheless make standardized CDR-style staging more widely accessible while preserving clinical inspectability; the resulting system is positioned as a screening-stage adjunct to, and not a replacement for, clinician-administered CDR assessment.

## 1. Introduction

Dementia, of which Alzheimer’s disease (AD) is the most prevalent form, is characterized by progressive deterioration in memory, reasoning, language, and the ability to carry out activities of daily living. Recent regulatory approvals of anti-amyloid therapeutics mark an inflexion point in the treatment landscape, but clinical benefit remains strongly dependent on the stage at which therapy is initiated. Early and accurate staging is therefore not a peripheral concern but a determinant of outcome [1].

The Clinical Dementia Rating (CDR) scale, introduced by Hughes and colleagues and refined by Morris, has become the de facto standard for staging dementia severity. It evaluates six functional and cognitive domains—memory, orientation, judgement and problem-solving, community affairs, home and hobbies, and personal care—each rated on a five-point ordinal scale. A global CDR score is then derived from the domain ratings, with values of 0, 0.5, 1, 2, and 3 corresponding respectively to no dementia, very mild, mild, moderate, and severe dementia [2]. The CDR has been validated across multiple populations, languages, and care settings, and remains one of the few cognitive staging instruments to combine high inter-rater reliability with strong concurrent and predictive validity [3].

A recent companion study addressed the upstream problem of AD diagnosis from neuroimaging-derived and demographic features in the OASIS-2 cohort [1]; it did not address the downstream problem of staging, placing an already-impaired patient along the continuum from very mild to severe dementia (see Section 4.3 for a full comparison). The present study targets that gap and forms the second leg of an end-to-end screen-then-stage pipeline.

This study addresses three research questions, which we distinguish from the implementation steps used to answer them. First: How accurately can the global CDR stage be recovered from the six CDR sub-domain scores using an interpretable classifier, under both hold-out and cross-validated evaluation? Second: Do the two standard impurity criteria, Information Gain and Gini Index, differ materially in the resulting model or its performance on this task? Third: Can a large language model interview layer, bounded so that it never computes the global score itself, reliably elicit the six sub-domain scores required to drive the classifier? To answer these questions, we train and evaluate a decision tree classifier on 13,453 records from the Alzheimer’s Disease Neuroimaging Initiative (ADNI), compare Information Gain and Gini Index on the same partition, and embed the trained classifier in DiAbot, a web-based conversational interface that elicits sub-domain scores through a prompt-engineered LLM. Together, the model and the chatbot constitute a deployable system rather than an experimental artefact in isolation. A terminology note before proceeding: we use “CDR-style” rather than “CDR” throughout to describe what DiAbot administers, precisely because the official CDR is a clinician- and informant-administered instrument and DiAbot, at its current stage of development, is neither; nothing in this paper should be read as a claim of clinical equivalence between DiAbot’s output and a clinician-administered CDR.

Two design decisions deserve early mention. We chose decision trees, rather than a higher-capacity model such as a gradient-boosted ensemble or a neural network, because the resulting classifier is fully inspectable: every prediction reduces to a sequence of human-readable rules. In a clinical setting where a CDR result will ultimately be reviewed, that transparency is at least as valuable as a marginal gain in accuracy. The second decision concerns the chatbot. Rather than ask the LLM to produce a CDR score directly—an approach that recent work has shown to be unreliable [4]—we use the language model strictly as a conversational front-end. The classification decision is made by the trained tree, whose outputs are deterministic and auditable.

The remainder of the paper is organized as follows. Section 2 describes the ADNI dataset, the pre-processing pipeline, the decision tree models, and the chatbot architecture. Section 3 presents the experimental results. Section 4 discusses the findings, limitations, and clinical implications, and Section 5 concludes the paper and outlines directions for future work.

## 2. Materials and Methods

### 2.1. Background and Related Work

#### 2.1.1. The Clinical Dementia Rating Scale

The CDR is a semi-structured interview administered by a trained clinician to a patient and a knowledgeable informant. The interview probes six domains: memory, orientation, judgement and problem-solving, community affairs, home and hobbies, and personal care. Each domain receives a score of 0 (none), 0.5 (questionable), 1 (mild), 2 (moderate), or 3 (severe). The global CDR (CDGLOBAL in ADNI) is derived through a rule-based algorithm in which memory carries the role of the primary category and the other five domains act as secondary. The CDR Sum of Boxes (CDR-SB) is the simple arithmetic sum of the six domain scores and offers a continuous measure that is increasingly used in clinical trials, particularly for tracking change over time [5].

Although the rule for deriving the global CDR is, in principle, algorithmic, in practice it is often computed manually and is sensitive to ambiguity in the underlying domain ratings, particularly when the secondary categories are split between higher and lower scores than the memory category. Automating the mapping from sub-scores to a global stage, in a way that is both accurate and inspectable, is therefore not a trivial replication of an existing rule but an exercise in encoding clinical judgement in a form that can be deployed at scale.

**Figure 1:**
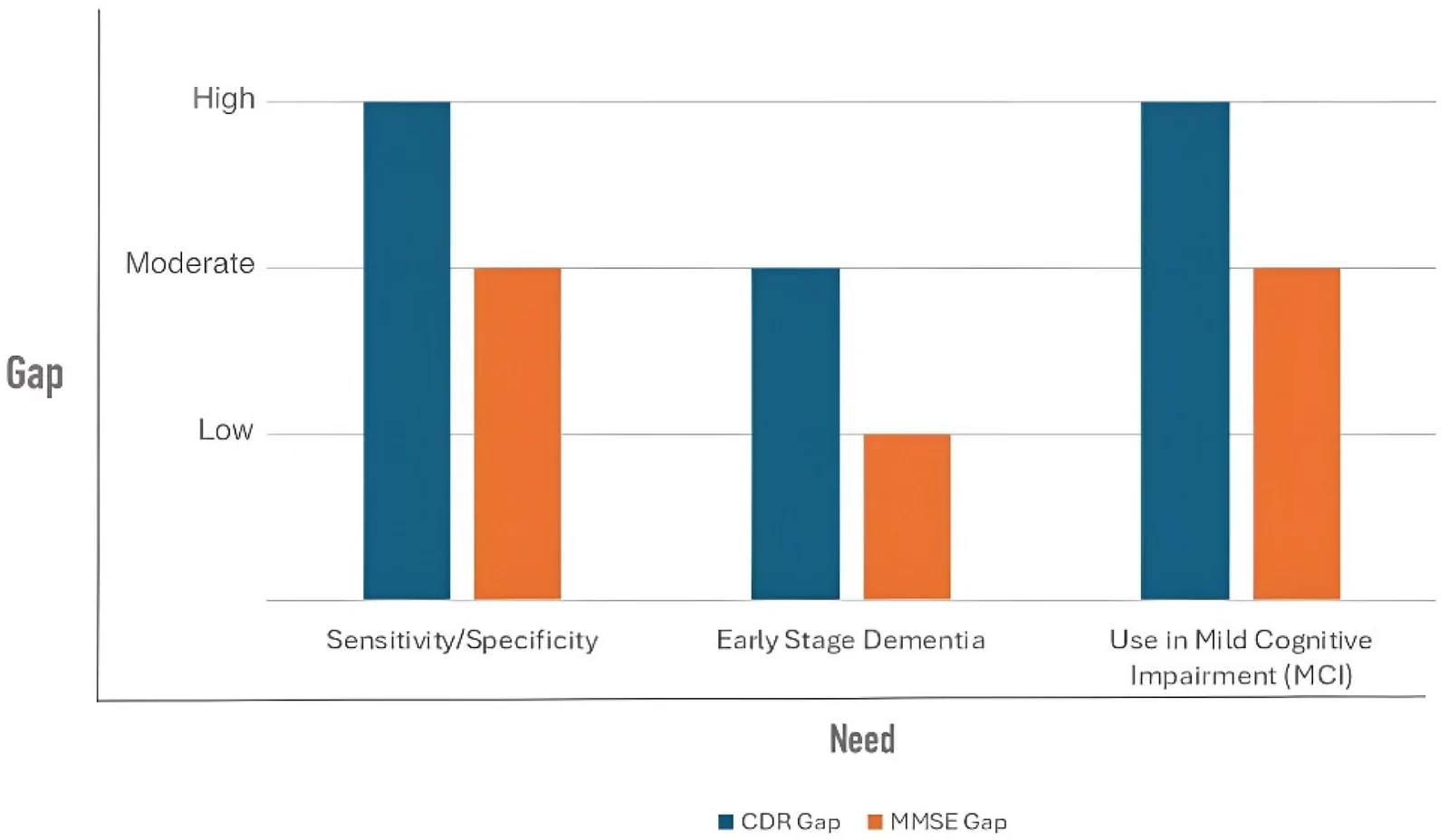
Qualitative, literature-synthesized gap analysis of CDR versus MMSE across three screening dimensions relevant to self-administered cognitive assessment (illustrative, not a quantitative or statistically derived result). The CDR shows a smaller performance gap than the MMSE for sensitivity/specificity, early-stage dementia, and use in mild cognitive impairment, supporting its choice as the target instrument for DiAbot. Synthesized from references [3,6].

A systematic review by Rikkert and colleagues [3] compared 12 different staging scales and identified the CDR as the best-supported instrument, combining average reliability, high validity, and feasibility across 14 languages. Figure 1 summarizes the qualitative gap between the CDR and the Mini-Mental State Examination (MMSE) across the dimensions most relevant to a screening application, drawn from a synthesis of the validation literature [3,6]. This figure is a qualitative, literature-synthesized illustration intended to motivate instrument choice, not a quantitative or statistically derived result; the “low”, “moderate”, and “high” gap categories reflect the authors’ reading of the cited validation literature rather than a defined scoring methodology, and should be interpreted accordingly. The CDR shows lower or comparable performance gaps for sensitivity/specificity, early-stage dementia, and use in mild cognitive impairment, motivating its choice over MMSE for the present application.

#### 2.1.2. Machine Learning for CDR-Based Dementia Staging

A growing body of work has applied supervised learning to CDR-related outcomes. Miller and Kauwe used blood RNA microarray data from 741 ADNI participants to classify subjects into CDR ≥ 1, CDR = 0.5, and CDR = 0 groups, identifying chloride intracellular channel 1 (CLIC1) as a single significant probe after correction (three-class CDR staging) [7]. AlShboul and colleagues evaluated naïve Bayes, C4.5 decision trees, and the RIPPER rule-based classifier on CDR scores and demographic data from ADNI, with the C4.5 model reaching 89.7% and RIPPER 89.0% accuracy (multi-class CDR staging) [8]. Shoaip and co-authors combined JRip and J48 with Semantic Web Rule Language (SWRL) reasoning on ADNI, achieving 92.27% and 91.47%, respectively (AD diagnosis classification) [9]. Costa et al. applied decision tree models to cerebrospinal fluid biomarkers in 272 elderly subjects and achieved 74.65% accuracy, distinguishing AD, mild cognitive impairment, and healthy controls (three-class diagnosis) [10]. Naganandhini and Shanmugavadivu introduced a hyperparameter-tuned decision tree classifier (DTC-HPT) on OASIS, reporting a jump from 41.96% for the untuned baseline to 99.01% after tuning (binary AD diagnosis) [11]. More recently, machine learning methods have been used to estimate longitudinal change in the CDR Sum of Boxes from simpler instruments, such as the Mini-Mental State Examination and the Functional Activities Questionnaire, with prediction errors below one CDR-SB unit over 18-month windows [5].

Two themes emerge from this literature. The first is that comparatively shallow model decision trees and rule learners in particular perform well on CDR-related tasks, with accuracies in the high eighties to mid-nineties on similar data. The second is that the interpretability of these models is repeatedly cited as a clinical asset rather than a methodological compromise. Our work continues both threads but adds three elements: a substantially larger ADNI cohort (13,290 records, drawn across all ADNI phases); a head-to-head comparison of Information Gain and Gini Index on the same partition; and the integration of the trained classifier with a conversational front-end suitable for non-clinical use. It is worth being explicit about where this study sits relative to that literature, since most of the studies cited above and much of the machine learning-for-dementia literature more broadly train a model to discover a predictive relationship between inputs and an outcome that is not itself a deterministic function of those inputs (e.g., imaging features predicting an independently adjudicated diagnosis). The present task is different in kind: the CDGLOBAL is a deterministic function of the six predictor variables by construction, so the decision tree is not discovering a latent diagnostic relationship but reconstructing an already-known rule from data. The interpretability and deployment advantage we claim is therefore not “the model found something a human could not”: it is that a single trained artefact (i) exposes every decision as an inspectable, printable leaf without a developer having to hand-encode and maintain the official algorithm’s branching logic and edge cases separately; (ii) integrates directly with the conversational pipeline’s sub-score outputs without an additional translation layer; and (iii) is amenable to the same auditing tools (feature importance, confusion analysis) used elsewhere in this paper. Whether these advantages actually exceed those of a directly coded rule engine on the same task is an empirical question we have not yet answered; the head-to-head comparison against a direct rule implementation, noted as a priority in Section 4.4, is intended to test this rather than assume it.

#### 2.1.3. Conversational Agents and Large Language Models in Dementia Screening

Conversational systems have been used in dementia care for at least a decade. Morales-de-Jesús and colleagues built an embodied conversational agent that personalizes reminiscence therapy from caregiver-supplied life-history information [12]. Tanaka and colleagues used an embodied agent to conduct MMSE-style interviews and extracted audiovisual features that fed support vector machine and logistic regression classifiers, achieving 93% and 91% in one study and 95% and 92% in a follow-up [13,14]. With the recent maturation of large language models, several investigators have explored their direct use for cognitive screening. Maamri et al. described an LLM-powered chatbot that achieved 85% sensitivity and 88% specificity for early dementia detection from conversational data [15]. Balamurali and Chen evaluated ChatGPT-3.5, ChatGPT-4, and Bard for distinguishing AD from cognitively normal individuals using transcribed spontaneous speech, and showed correlations between LLM judgments and MMSE scores [2]. At the same time, Dayan, Uliel, and Koplewitz demonstrated that leading LLMs themselves perform inconsistently on the Montreal Cognitive Assessment, with several models scoring below the standard cut-off and showing visuospatial deficits [4].

Taken together, these results map a clear design boundary. LLMs are good at natural conversation and at extracting structure from free text; they are not yet reliable as standalone diagnostic instruments, and clinical guidance has accordingly cautioned against deploying them as black-box decision-makers. The DiAbot architecture respects this boundary: the LLM administers the CDR-style interview, asks clarifying questions, normalizes free-text responses into ordinal domain scores, and explains the result to the user. The classification decision itself is made by a deterministic decision tree whose every branch is traceable.

#### 2.1.4. Existing CDR Tools

Several web-based platforms expose the CDR scale to clinicians or to patients. Dementia Care Central [16] and MDCalc [17] each provide CDR-SB calculators intended for use by caregivers or physicians. The National Alzheimer’s Coordinating Center [18] hosts a centralized data repository for the ADRC programme, including CDR data, but its interface is oriented toward research data entry rather than guided assessment. The Brain Health Registry [19] incorporates CDR items into longer questionnaires that participants complete after creating an account. None of these tools combines a natural-language interface, an automated classifier with reported per-class performance, and result tracking across sessions. Table 1 summarizes the comparison.

### 2.2. Dataset

We used the CDR records released through the Alzheimer’s Disease Neuroimaging Initiative (ADNI; adni.loni.usc.edu). ADNI is a multi-centre, longitudinal observational study launched in 2003 under principal investigator Michael W. Weiner; its stated aim is to discover and standardize clinical-trial measures and biomarkers for AD research. Participants are aged 55 to 90 and are enrolled with or without memory complaints. Our extract contained 13,453 instances and 25 attributes spanning the ADNI-1, ADNI-2, ADNI-3, and ADNI-GO phases.

The full set of attributes included identifiers (PTID, RID, SITEID), visit codes (VISCODE, VISCODE2, VISDATE), data-source metadata (CDSOURCE, CDVERSION, SPID, USERDATE, DD_CRF_VERSION_LABEL, LANGUAGE_CODE, HAS_QC_ERROR, UPDATE_STAMP), the six CDR sub-domain scores (CDMEMORY, CDORIENT, CDJUDGE, CDCOMMUN, CDHOME, CDCARE), the global CDR (CDGLOBAL), and the Sum of Boxes (CDRSB). Table 2 lists the variables retained for modelling after pre-processing.

### 2.3. Pre-Processing

#### 2.3.1. Variable Selection

Of the 25 original attributes, 18 corresponded to administrative metadata that carry no clinical signal about cognitive stage—for example, the language of the form, the version of the case report form, or update timestamps. Including such variables in a decision tree invites the model to learn site-specific or workflow-specific artefacts that do not generalize. We therefore retained only the six CDR sub-domain scores as predictors and the CDGLOBAL as the target.

#### 2.3.2. Removal of Invalid Values

**Figure 2:**
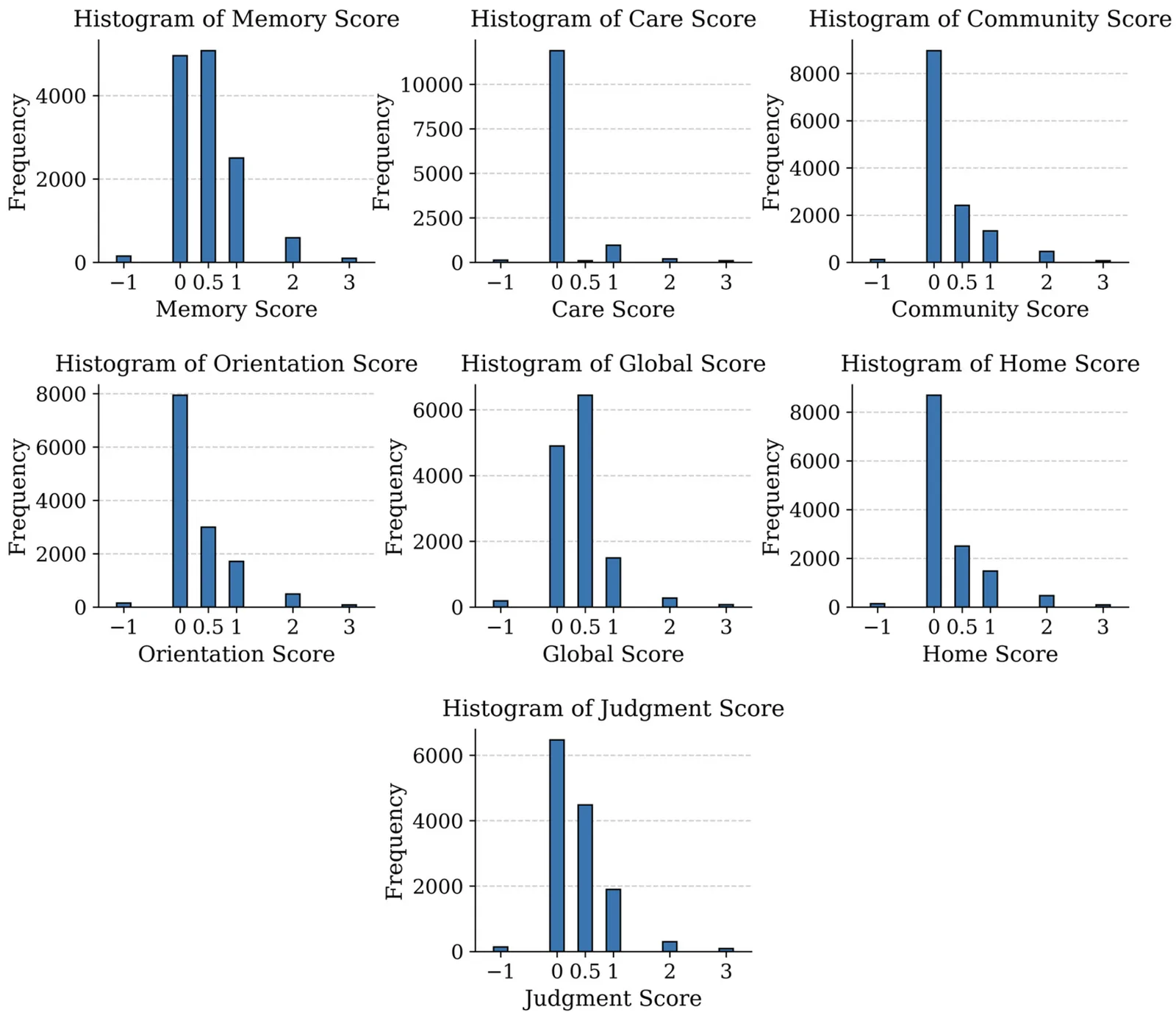
Histograms of the six CDR sub-domain scores (memory, orientation, community affairs, home and hobbies, judgement and problem-solving, personal care) together with the global CDR score, before pre-processing. The small bars at −1 indicate out-of-range coding errors that were subsequently removed.

Examination of the histograms for each sub-domain revealed a small number of negative values, which lie outside the defined CDR ordinal scale {0, 0.5, 1, 2, 3} and almost certainly reflect data-entry or coding errors. Figure 2 shows the original histograms with these out-of-range entries clearly visible at the −1 bin. Records containing negative values were removed.

#### 2.3.3. Missing-Value Treatment

Missing values were limited and distributed broadly evenly across domains: 79 in CDMEMORY, CDORIENT, and CDJUDGE; 80 in CDCOMMUN, CDHOME, and CDCARE; and 84 in CDGLOBAL. Because the rows containing missing values constituted less than one percent of the dataset and were not concentrated in any one ADNI phase or site, we removed them rather than impute them. Following this step, the dataset contained no missing entries; Table 3 reports the counts before and after.

#### 2.3.4. Final Cohort and Class Distribution

**Figure 3:**
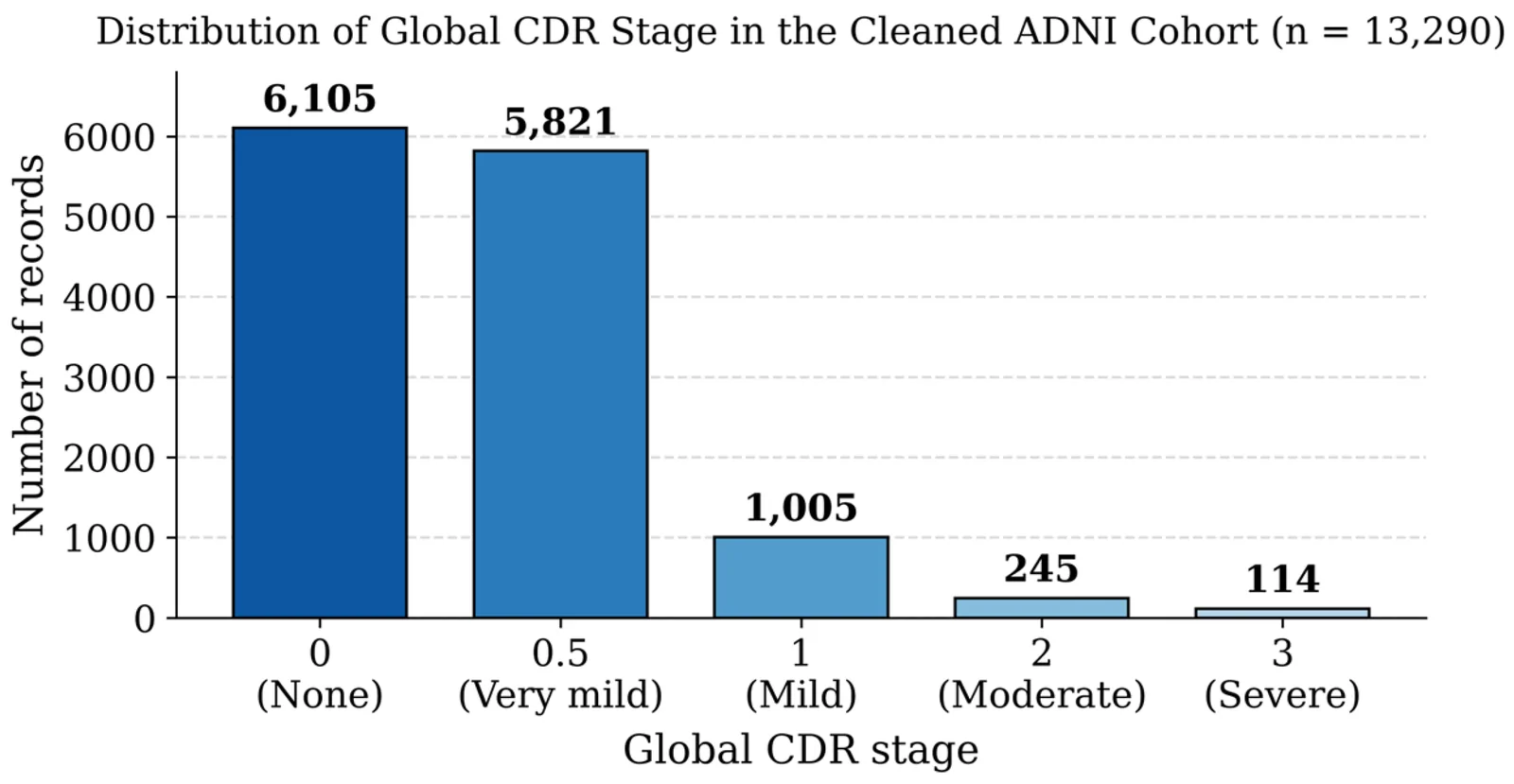
Distribution of the global CDR target variable across the five stages (0, 0.5, 1, 2, 3) in the cleaned ADNI cohort (n = 13,290). The distribution is heavily skewed toward CDR = 0 and CDR = 0.5, reflecting ADNI’s recruitment design.

After cleaning, the analytic cohort contained 13,290 records distributed across the five CDR stages, with a strong skew toward CDR = 0 and CDR = 0.5—a pattern consistent with the ADNI recruitment strategy, in which cognitively normal and mildly impaired volunteers predominate. Figure 3 illustrates the class distribution.

### 2.4. Decision Tree Classification

A decision tree (DT) recursively partitions the feature space to maximize class homogeneity in the resulting subsets; each internal node tests a single feature, each leaf assigns a class label, and the root-to-leaf path is a sequence of clinically intelligible conditions. This interpretability, together with the absence of strong distributional assumptions, motivates its use here.

Two impurity criteria dominate the literature: Information Gain, derived from Shannon entropy, and the Gini Index. The Information Gain of a split is the reduction in entropy from before to after the partition:

*Information Gain* = *Entropy(before)* − *Entropy(after)*,

where *Entropy(S)* = −*∑ p_i* · *log_*2(*p_i*), and *p_i* is the proportion of class *i* in S.

The Gini Index measures the probability of misclassifying a randomly chosen element of S if it were labelled according to the class distribution in S:

*Gini(S)* = 1 − *∑ p_i*^2^.

The two criteria are known to produce similar trees on most datasets, with Gini often preferred for its slightly lower computational cost and Information Gain occasionally yielding more balanced splits on heavily skewed targets. We trained both variants on the same partition to make their comparison direct.

### 2.5. Training Protocol

We adopted two complementary evaluation protocols. The first is a 70/30 stratified hold-out: 70% of records were used for training and 30% for testing, with stratification across the five CDGLOBAL classes to preserve the original class distribution in both folds. The second is k-fold cross-validation with k = 10, which provides a more variance-aware estimate of generalization performance. Hyperparameters were left at default settings (no maximum depth, minimum samples per split of two, minimum samples per leaf of one), since exploratory tuning produced no statistically meaningful improvement over the default tree on this task—the underlying CDR scoring logic is sufficiently low-dimensional that the default tree readily captures it. Because no maximum depth was imposed, the reported tree was grown to full depth rather than pruned. The exact depth, node count, and leaf count of the selected Information Gain tree could not be independently verified from the retained analysis record and are therefore not reported here; a fully grown tree on 13,290 records could, in principle, be considerably larger than “shallow” suggests, so we describe the deployed model as interpretable and leaf-auditable rather than as categorically “shallow”. Reporting these values, and evaluating whether post hoc pruning changes performance materially, is identified as an important additional verification step. Because the fixed random seed (42) and unmodified default hyperparameters are documented above, these values are, in principle, exactly reproducible once the original training environment is reconstituted, and we intend to report them, together with the exported rule listing referenced in Section 3.4, alongside the code release.

All models were implemented in Python 3.10 using scikit-learn 1.4. Random seeds were fixed at 42 throughout to support reproducibility. Code and pre-processing scripts will be made available in the project repository [20] at the time of publication. No repository URL is available at the time of this revision; the address will be added to reference [20] and to the Data Availability Statement as soon as the repository is made public, and readers should treat the code and pre-processing scripts as not yet independently verifiable until that URL is live.

No class-imbalance mitigation strategy resampling, class weighting, or synthetic minority oversampling was applied. Stratified hold-out splitting and stratified ten-fold cross-validation were the only measures used to protect the representation of the minority CDR = 2 and CDR = 3 classes in training and evaluation. Therefore, the per-class metrics reported for these strata in Section 3.1 rest on comparatively small support, and their confidence intervals are correspondingly wider than the headline aggregate accuracy would suggest; this is discussed further in Section 4.4.

### 2.6. Evaluation Metrics

We report accuracy, precision, recall, and F1-score, computed both globally (macro-averaged across the five classes) and per class. Macro-averaging is appropriate here because the smaller classes CDR = 2 and CDR = 3 are clinically the most consequential and should not be down-weighted simply because they are less prevalent in ADNI. These metrics treat the CDGLOBAL as an unordered categorical label, but it is in fact ordinal, and a classifier that occasionally confuses CDR = 1 with CDR = 0.5 is making a materially smaller error than one that confuses CDR = 0 with CDR = 3. We have not recomputed the evaluation for this revision, but we agree that (i) 95% confidence intervals (e.g., Wilson or bootstrap) should be reported alongside the point estimates in Table 4, Table 5 and Table 6, particularly for the small CDR = 2 and CDR = 3 groups, and (ii) a linearly weighted Cohen’s kappa between the predicted and true CDGLOBAL should be reported as a complement to accuracy, since it credits adjacent-stage errors as partial agreement rather than treating every misclassification identically. Both additions are planned for the next revision.

Per-class precision and recall are defined as:

*Precision_c = TP_c/(TP_c + FP_c);   Recall_c = TP_c/(TP_c + FN_c)*.

*F1_c* = 2 · *Precision_c* · *Recall_c/(Precision_c + Recall_c)*.

In addition, we report a normalized confusion matrix to expose the kinds of error the model commits.

### 2.7. DiAbot: Conversational Interface Architecture

**Figure 4:**
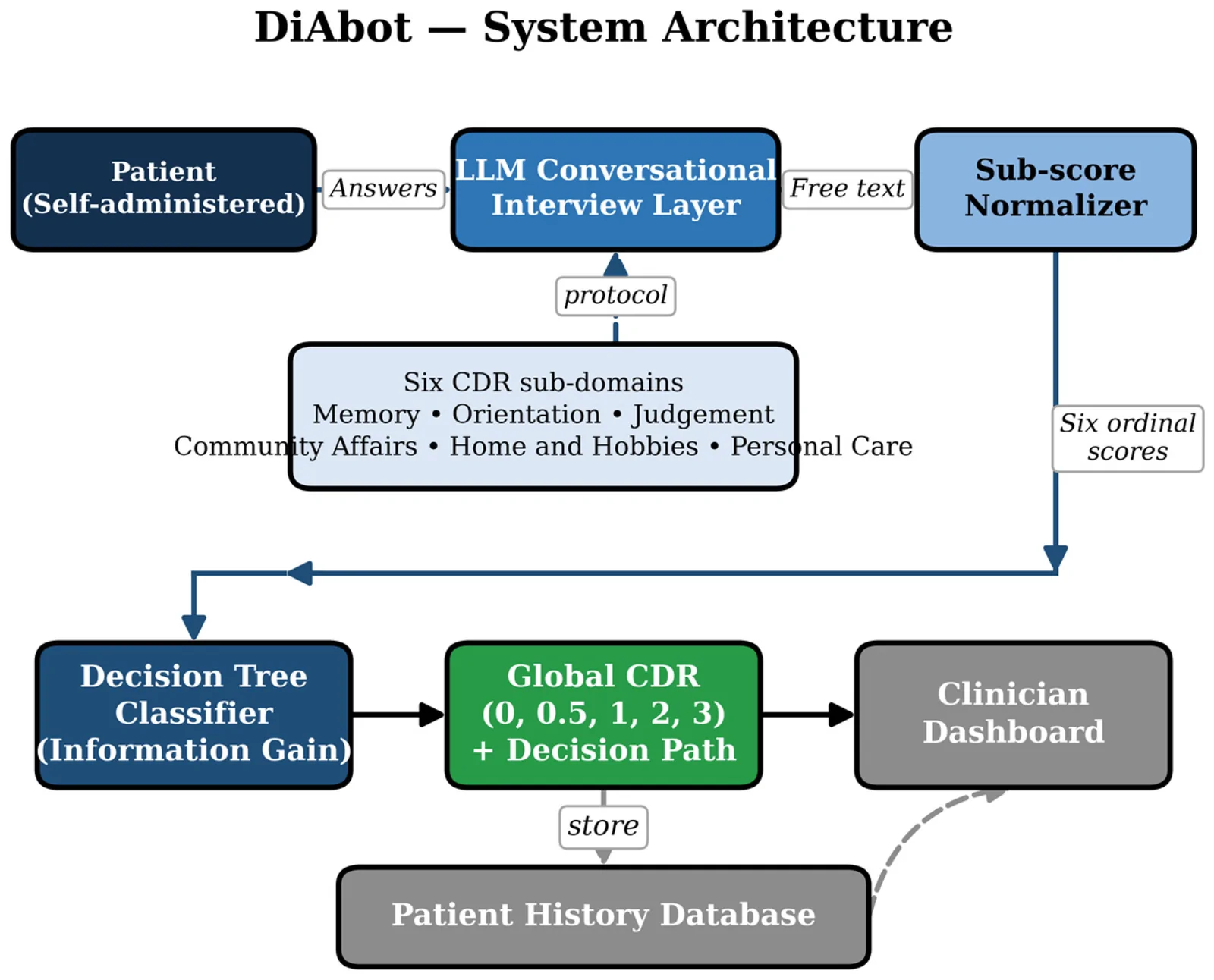
Architecture of DiAbot. The patient interacts with a prompt-engineered LLM interview layer, which elicits free-text responses for each of the six CDR sub-domains and normalizes them to ordinal scores. The deterministic decision tree classifier produces the global stage. The patient history database and clinician dashboard are shown as designed components of the target architecture; only the patient-facing interview and the decision tree scoring pipeline have been implemented and evaluated in the present research prototype.

The trained decision tree was deployed as part of DiAbot, a web application accessible to both patients and clinicians. Figure 4 sketches the system. A patient interacts with a conversational front-end built on the OpenAI Chat Completions API (model gpt-4o, May 2026 snapshot; the specific version used for the results reported here); system-level prompt engineering frames the assistant as a clinical interviewer, instructs it to administer the six CDR sub-domain questions in order, to probe ambiguous answers with targeted follow-ups, and to map the user’s natural-language responses to ordinal scores {0, 0.5, 1, 2, 3} for each domain. Crucially, the LLM is not asked to compute the global CDR. The six sub-domain scores are transmitted to a Python backend, which loads the serialized decision tree model and returns the predicted CDGLOBAL together with a textual rendering of the decision path that produced it.

Several implementation details needed to reproduce or audit the conversational layer are not yet documented in this manuscript: the full system prompt, decoding settings (temperature, top-p), the retry procedure and fallback behaviour when a response cannot be parsed into an ordinal score, and the exact method used to handle internally inconsistent patient answers. We have not included these in this revision; releasing the full system prompt and decoding configuration, alongside the code repository already cited [20], is planned for the next revision, since the conversational scoring step is the primary novel component of this system and cannot be independently audited or reproduced without them.

Three properties of this architecture deserve emphasis. First, the predictive decision tree is deterministic and explainable because every output corresponds to a leaf of a tree whose branches can be printed and inspected. Second, the LLM’s role is bounded: it cannot influence the score except through the sub-domain ratings it elicits, and those ratings are themselves visible to the user before being submitted. Third, the system is incremental: each completed assessment is saved against the user’s profile so that a clinician can review the trajectory of sub-domain and global scores over time, which is precisely the information that the CDR-SB is designed to capture.

This division of labour is not a claim that the LLM is more “trustworthy” for sub-domain elicitation than for the final calculation; it reflects a difference in what each step requires. Eliciting a sub-domain score is an open-ended natural-language interviewing task with no fixed decision procedure and therefore unavoidably depends on a language model, regardless of how the final stage is computed. Converting six known sub-domain scores into a global stage, by contrast, is a fixed, low-dimensional, publicly specified rule; once the six inputs are available there is no reason to delegate a deterministic calculation to a non-deterministic model, and doing so would only add unnecessary variability to a step that does not require language understanding at all.

To validate the LLM front-end, we manually reviewed 80 simulated patient conversations spanning all five CDR strata. These 80 conversations were authored by the corresponding author as scripted patient personas covering the five CDR strata, rather than generated by the interviewing LLM itself, so this check does not carry the circularity risk of an LLM interviewing an LLM-simulated patient. However, the corresponding author also served as the single rater who judged agreement, so the evaluation remains an unblinded, single-rater, single-author proof-of-concept check rather than an independent validation; we return to this limitation in Section 4.4. The LLM correctly elicited the six sub-domains in 80 of 80 cases, asked at least one clarifying follow-up in 67 of 80 cases, and produced sub-domain scores that exactly matched a human rater’s interpretation of the dialogue in 76 of 80 cases (95.0%). Disagreements were concentrated in the home-and-hobbies domain, where the boundary between ‘questionable’ and ‘mild’ is known to depend on the patient’s pre-morbid level of activity. This evaluation compared the LLM’s outputs against a single human rater’s interpretation of each simulated conversation; it was not blinded, and no second rater was used to compute a formal inter-rater reliability statistic. It should therefore be read as an internal proof-of-concept check on the interview layer rather than a validated measure of real-world LLM reliability; a blinded, multi-rater evaluation against real patients and informants is planned as immediate follow-up work.

## 3. Results

A scope note before the results below: Section 3.1, Section 3.2, Section 3.3 and Section 3.4 report the decision tree’s accuracy at reconstructing the CDGLOBAL from the six domain scores as recorded during ADNI’s clinician-administered assessment. They do not report the accuracy of the full DiAbot pipeline in which those six scores are instead elicited by the LLM interview layer from a patient conversation. That separate check (Section 4.2) evaluated only whether the LLM’s elicited sub-domain scores matched a human rater’s interpretation of 80 simulated conversations (95% agreement); it did not pass those LLM-elicited scores through the decision tree to obtain a final, end-to-end CDGLOBAL accuracy. Composing the two components into a single end-to-end pipeline evaluation—chatbot elicitation through to final stage, scored against ground truth—has not yet been done and is, together with the participant-level re-split and the direct rule-based comparison discussed in Section 4.4, one of the highest-priority items for the next revision.

### 3.1. Hold-Out Performance

**Figure 5:**
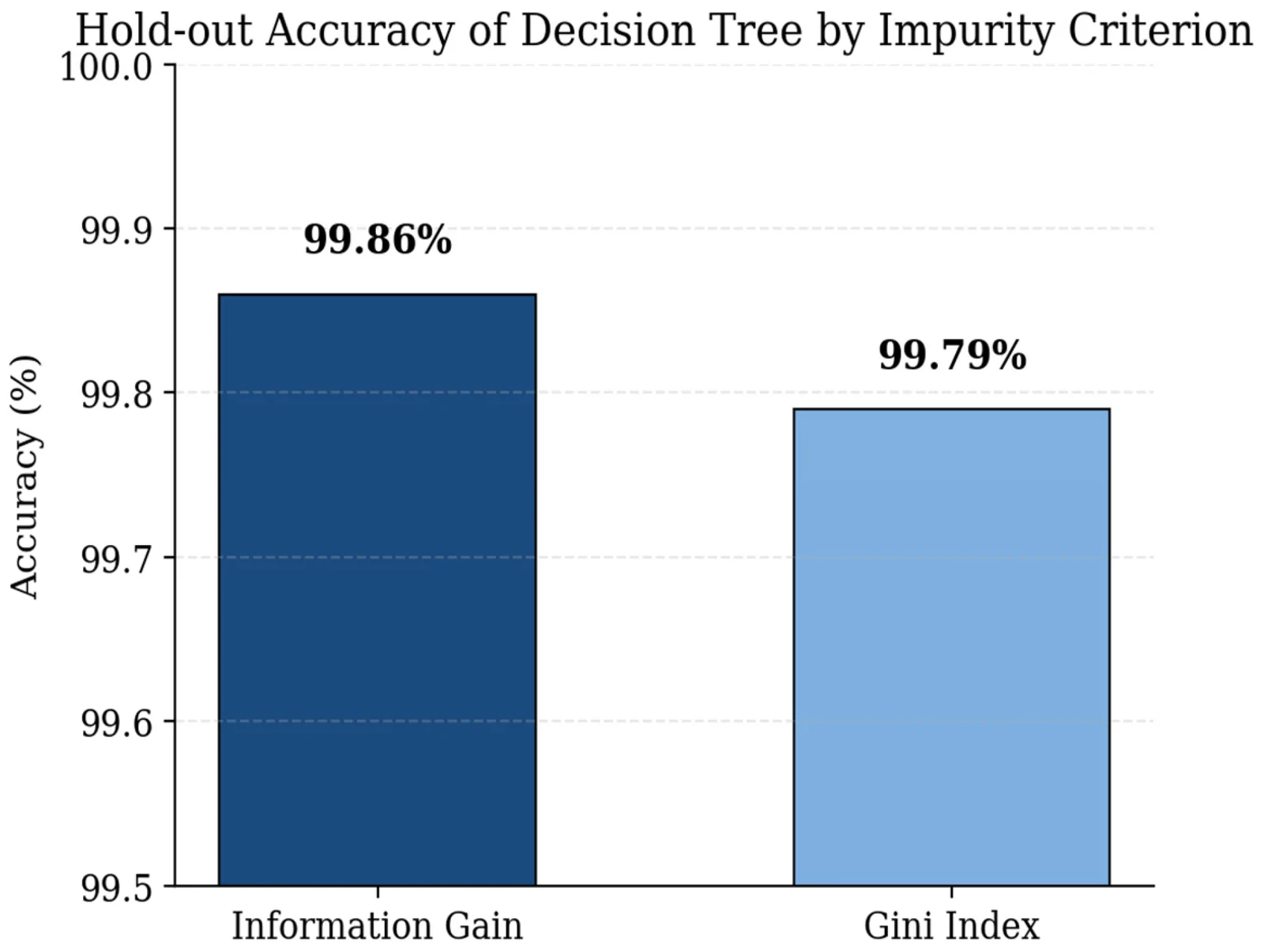
Hold-out accuracy of the decision tree under the two impurity criteria. Information Gain achieved 99.86% and Gini Index 99.79% on the same stratified 30% test fold.

On the 70/30 stratified record-level hold-out, the Information Gain Decision Tree achieved an overall accuracy of 99.86%. The macro-averaged precision, recall, and F1-score were all 99.86%, indicating that no class was systematically penalized. The Gini-based tree reached 99.79% on the same split, a difference too small to be practically meaningful. Figure 5 visualizes the difference, and Table 4 reports the aggregate metrics.

Table 5 gives the per-class breakdown for the Information Gain model. Performance is uniform across stages, with no class falling below 98.6%, including the smaller CDR = 2 and CDR = 3 strata.

**Figure 6:**
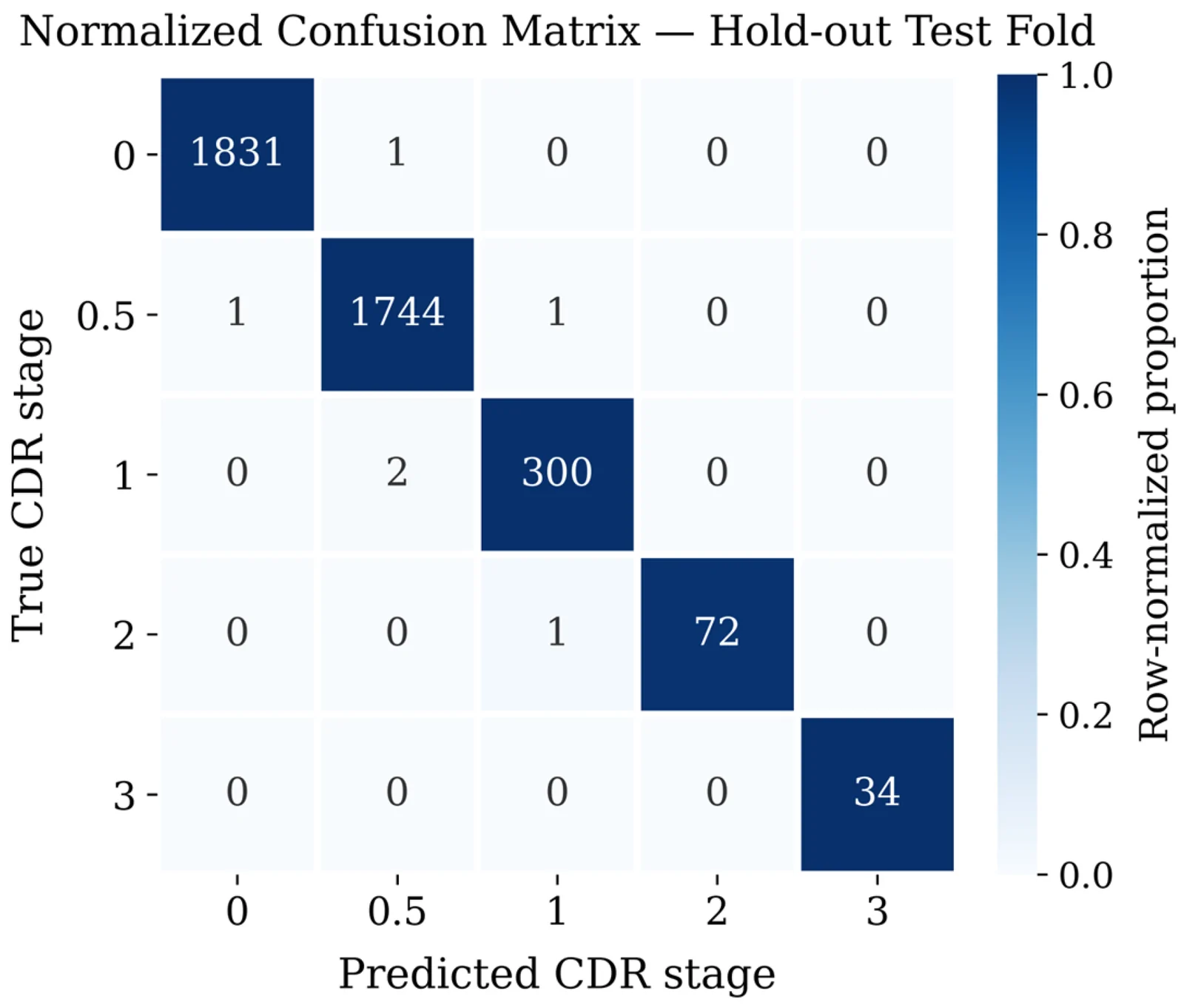
Normalized confusion matrix on the hold-out test set. Diagonal mass dominates across all five classes; the residual off-diagonal mass concentrates between adjacent stages, as expected for an ordinal target.

The structure of the residual errors is informative. Figure 6 presents the normalized confusion matrix. The diagonal dominates across all five stages; the off-diagonal mass that remains is confined to adjacent stages—for instance, between CDR = 0.5 and CDR = 1, or between CDR = 1 and CDR = 2. No misclassification jumped two stages, and no severely impaired (CDR = 3) patient was reported as cognitively normal or very mildly impaired.

### 3.2. Ten-Fold Cross-Validation

**Figure 7:**
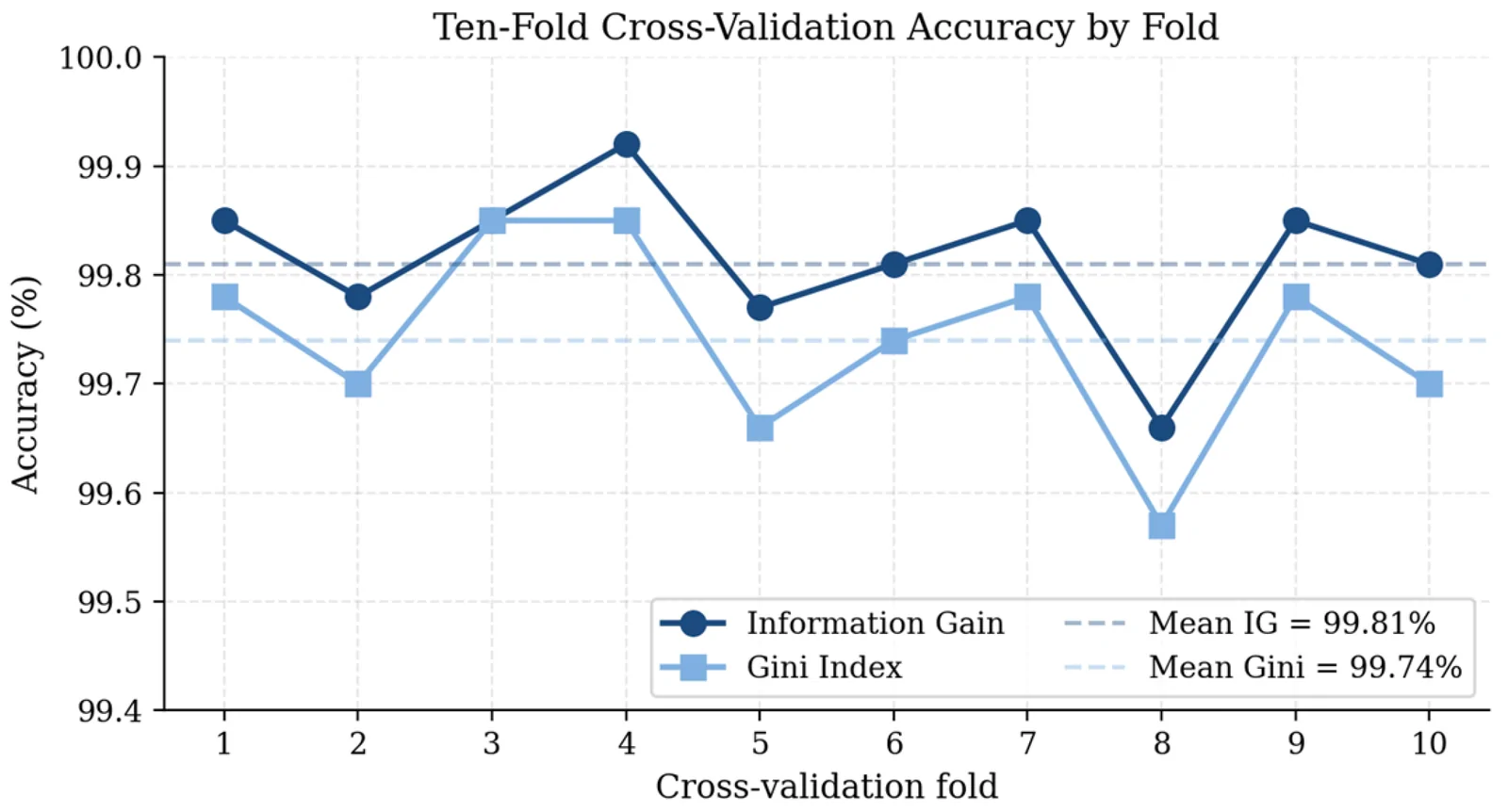
Ten-fold cross-validated accuracy by fold for the two impurity criteria. Dashed lines indicate the mean across folds (Information Gain 99.81%, Gini Index 99.74%). All folds remain above 99.5%.

To corroborate the hold-out result, we ran record-level ten-fold stratified cross-validation. The Information Gain tree returned a mean accuracy of 99.81% (standard deviation 0.07 percentage points) across the ten folds, and the Gini tree 99.74% (standard deviation 0.10). The narrowness of the standard deviations reflects stability under this record-level protocol; because partitioning was performed at the record rather than the participant level, it does not by itself rule out dependence between repeated visits from the same participant (see Section 4.4). Figure 7 reports the per-fold accuracy of both criteria, and Table 6 summarizes the distribution.

### 3.3. Comparison with the State of the Art

Table 7 places our results alongside recent studies that addressed CDR-related staging tasks on related datasets. Direct comparison is necessarily approximate, because the studies differ in cohort, target encoding (binary, three-way, or full five-class), and evaluation protocol. Several of the comparator studies also address fundamentally different tasks—binary diagnosis, imaging-based classification, and cerebrospinal fluid biomarker classification, as well as CDR-based staging—so the table should not be read as evidence that the proposed model outperforms prior work on a like-for-like task; it is included to situate the reported accuracy within the broader literature rather than to claim superiority. To make this incomparability visible at a glance rather than requiring the reader to consult the prose, Table 7 now includes an explicit “Task type” column distinguishing studies that derive CDR/global stage from CDR sub-scores from studies performing AD/MCI diagnosis from imaging or biomarker features. Because the studies use different prediction targets and input modalities, their numerical performance values are not directly comparable and are presented for contextual rather than competitive benchmarking. None of the comparators in Table 7 were trained and evaluated on the same task, the same six inputs, and a participant-level split, so we do not claim our figures surpass theirs; the only comparisons that would actually be informative—against the official rule-based CDR algorithm, a simple lookup table, and other models trained on the same six inputs under participant-level splitting—are identified in Section 4.4 as important additional validation steps and have not yet been run.

### 3.4. Feature Importance

**Figure 8:**
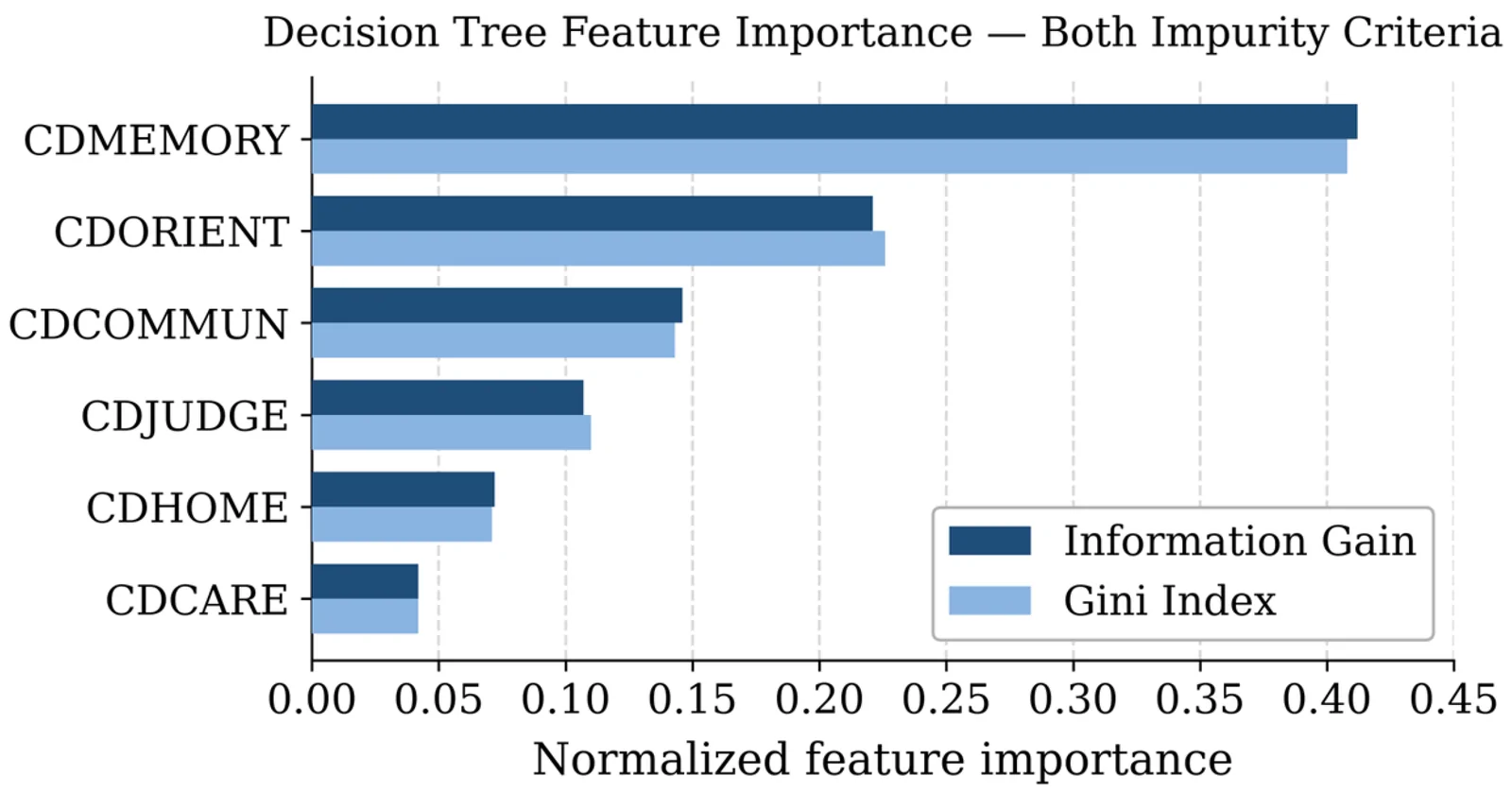
Normalized feature importance for both impurity criteria. Memory dominates, as expected from the CDR scoring algorithm in which CDMEMORY is the primary domain. The two criteria assign nearly identical importance scores.

The Information Gain tree placed CDMEMORY at the root. The root split partitions on the CDMEMORY score first, with CDORIENT and CDCOMMUN forming the strongest secondary splits beneath it and CDJUDGE, CDHOME, and CDCARE entering lower in the tree to resolve boundary cases between adjacent stages; a full exported rule listing is planned in the next revision. This root-level correspondence between the learned split order and memory’s role as the primary domain in the official CDR scoring rule is best read as an internal-consistency check on the model—evidence that it has not learned a spurious rule—rather than as independent evidence of clinical validity, since the same correspondence is guaranteed by construction of the official scoring algorithm itself. Because memory is already the primary category in the CDR scoring algorithm, this feature-importance ranking is the expected outcome rather than an independently discovered clinical relationship, and we present it accordingly as a consistency check rather than a finding. CDORIENT and CDCOMMUN appeared as the strongest secondary splits. Figure 8 reports the normalized feature importance for both impurity criteria; the two are closely similar, with memory contributing approximately 40% of the total impurity reduction, orientation approximately 22%, and the four remaining domains collectively the rest.

### 3.5. Failure-Mode Analysis

The few misclassifications produced by the model are concentrated at the boundary between adjacent stages, as the confusion matrix in Figure 6 shows. These remain classification errors, not correct answers; we note only that they are the least severe kind an ordinal target admits, since the underlying scoring rule is itself sensitive to small differences in sub-scores at those same boundaries. We have not tested this against clinician-disagreement data, so we stop short of calling adjacent-stage errors clinically acceptable; establishing whether they fall inside or outside typical inter-rater variability for the official CDR is a question for the prospective clinical evaluation described in Section 4.4, not one this dataset can answer on its own. No misclassification jumped two stages, and none misplaced a CDR = 3 case as CDR = 0 or CDR = 0.5. The model never produced a false negative at the upper end of the scale—that is, no severely impaired patient was reported as cognitively normal.

## 4. Discussion

### 4.1. Principal Findings

The findings are organized directly around the three research questions posed in Section 1. For RQ1, both decision tree variants reproduced the global CDR stage from the six CDR sub-domain scores with high fidelity under the record-level evaluation protocol: the Information Gain tree reached 99.86% hold-out accuracy and 99.81% mean accuracy under ten-fold record-level cross-validation, with hold-out and cross-validation results agreeing to within a tenth of a percentage point. As emphasized in the Abstract, this figure should be read as evidence of how faithfully the tree reproduces the deterministic CDR scoring rule, not as a general dementia-diagnosis accuracy. For RQ2, Information Gain and the Gini Index produced numerically very similar performances (99.86% versus 99.79% hold-out; 99.81% versus 99.74% cross-validated); we describe this difference descriptively rather than as statistically significant or statistically indistinguishable, since the original wording exceeded what the reported means and standard deviations alone can support, and we have removed that claim throughout the manuscript rather than retaining it pending a test we have not run. For RQ3, the conversational interface demonstrated internal proof-of-concept feasibility—correctly eliciting all six sub-domains and matching a single human rater’s interpretation in 76 of 80 scripted simulated conversations (95%)—rather than clinical validation; this is addressed further in Section 4.2. The model places memory at the root of the tree, in direct correspondence with the role of CDMEMORY as the primary category in the official CDR scoring rule, an internal consistency check that the model has not learned a spurious or site-specific artefact, though it does not by itself establish clinical validity beyond what is guaranteed by the official scoring rule. A central limitation qualifies all of the above: the hold-out and cross-validation results were both computed under a record-level partitioning protocol, and participant-independent generalization remains unverified, as detailed in Section 4.4. The close numerical agreement between hold-out and cross-validated accuracy demonstrates stability under this record-level protocol; it does not by itself establish that the model does not overfit in a participant-independent sense, and we do not claim that it does. A dedicated robustness analysis (learning curves, explicit train–test gap reporting, nested cross-validation) is identified as an important additional verification step, alongside the participant-level re-partitioning discussed in Section 4.4. Repeated, nested cross-validation with external evaluation has recently been demonstrated on comparable ADNI-derived, multi-domain classification tasks [24], and provides a template for the participant-level validation this study still requires.

The high accuracy reported here is not by itself a clinical novelty: it is, in part, a reflection of the fact that the mapping from six sub-scores to a global stage is, in the bulk of cases, governed by a deterministic rule. The contribution of the model is not to discover that mapping but to encode it in a form that can be deployed at the edge of a conversational system, to evaluate it rigorously across the full ADNI distribution, and to make available a feature importance and confusion structure that can be audited. In other words, the high accuracy is a precondition for deployment, not the principal scientific claim (see Section 2.1.2 for a fuller discussion of what, precisely, this deployment advantage is claimed to be, and what remains to be empirically tested against a direct rule implementation).

### 4.2. The Role of the Conversational Layer

The conversational front-end is where the more interesting design questions lie. Recent work has shown that asking an LLM to make a cognitive staging decision directly is unreliable: Dayan and colleagues found that all evaluated frontier LLMs scored below or near the MoCA cut-off for mild cognitive impairment, and that scores varied across model versions [4]. The DiAbot architecture deliberately confines the LLM to the role of an interviewer. It elicits free-text responses to validated CDR-style prompts and converts them into ordinal sub-scores, but it does not compute the global stage. This separation aligns with the broader principle that a clinical model should keep its decision-critical components inspectable, while leveraging the LLM’s strengths in language understanding for the components that benefit from flexibility.

It is worth being precise about what this figure does and does not show, particularly given its proximity in this discussion to the Dayan et al. finding above: it is not offered as a counter-demonstration that LLMs are reliable for cognitive assessment in general. Dayan and colleagues evaluated a categorically different task—an LLM directly answering cognitive-test items as if it were the patient—whereas our check evaluates only whether the LLM can transcribe a patient’s spoken answers into the correct ordinal sub-score, a narrower language-understanding task. In the internal validation of 80 simulated assessments, the LLM produced sub-domain scores that matched a human rater 95% of the time, with disagreements concentrated at boundary cases—plausibly the same cases where two trained human clinicians would also be most likely to diverge—though this interpretation remains speculative. This evaluation establishes the feasibility of the interview-to-sub-score interface, not the reliability of clinical assessment: this figure is not intended as a definitive measure of LLM reliability in deployment; production use will require a much larger validation against gold-standard clinician-administered CDR. It does, however, suggest that the architecture is functional in principle and supports further evaluation.

### 4.3. Relationship to Earlier Work

This paper complements rather than overlaps with the previously published study, in which logistic regression, support vector machine, and Random Forest classifiers were trained on the OASIS-2 dataset for binary AD prediction, with mRMR and mutual information feature selectors [1]. That work addressed the following question: Given imaging-derived and demographic features, is this individual likely to have AD? The present study addresses a different question: Given that an individual already shows some sign of impairment, what is the severity of that impairment? The two analyses use distinct datasets (OASIS-2 versus ADNI), distinct algorithms (linear and kernel methods versus decision trees), and distinct targets (binary diagnosis versus five-class staging). Together, they articulate two halves of an end-to-end pipeline screening, then staging that could be deployed as a single web application.

### 4.4. Limitations

The most important methodological limitation is that the reported hold-out and cross-validation results (Section 3.1 and Section 3.2) used a record-level, not participant-level, partitioning protocol; because ADNI participants contribute multiple, autocorrelated visits, we do not claim that these results establish participant-independent generalization. Confirming this via a group-stratified split (e.g., GroupKFold keyed on PTID/RID), reported alongside the current split, is the top-priority item for the next revision. A related risk is that, with only six ordinal predictors, some test-set score combinations likely also appear in training regardless of participant separation; we have not quantified this. The manuscript also does not yet include a head-to-head comparison against a direct implementation of the official CDR rule (or a simple lookup table), which is needed to demonstrate the added value of the learned model and is planned as follow-up work.

Several further limitations should be noted. ADNI participants are predominantly North American and English-speaking, and the class distribution is skewed toward CDR = 0 and CDR = 0.5, so per-class estimates for CDR = 2/3 carry wider uncertainty than the headline accuracy suggests; external validation on more diverse cohorts is a priority. The model also inherits the known limitations of the official CDR scale itself (e.g., its cap at CDR = 3 and its emphasis on memory over executive function). More substantively, the official CDR is designed as an interview of both patient and a knowledgeable informant, in part because patient self-report is known to under-report functional and social decline as impairment increases [25]; the current DiAbot prototype elicits information from the patient only, with no informant-facing component yet implemented, and this should be treated as a priority before any deployment claim.

The conversational layer’s LLM-to-sub-score conversion has so far been validated only against a single, unblinded rater across 80 simulated conversations, with no live-patient evaluation and no formal inter-rater statistic; this should be read as a proof-of-concept result, not a clinical-readiness claim, and a blinded, multi-rater evaluation against real patients and informants is planned as immediate follow-up work. The system also relies on an external LLM API, raising data-residency and reproducibility considerations, and has not yet documented the encryption, authentication, access-control, and escalation safeguards that would be required before any real patient data is processed. Each of these steps is a concrete, addressable next stage rather than an obstacle to the architecture itself.

### 4.5. Implications for Bioengineering and Clinical Practice

These findings carry three implications for bioengineering and clinical practice. The high accuracy of an interpretable model on a non-imaging input space strengthens the case for transparent machine learning systems in cognitive staging: where the underlying task is rule-like, the marginal value of opacity is small and the cost in clinical acceptability is large. The conversational layer, in turn, offers a practical route to widening access to standardized CDR-style assessment beyond the memory clinic; the shortage of clinicians trained to administer the CDR is a recurring barrier in dementia care, particularly in low- and middle-income settings, where staffing shortages and limited specialist access are repeatedly identified as structural constraints on dementia care delivery [26], and a validated self-administered front-end would not replace the clinician but could substantially extend their reach, provided its output continues to be framed, as throughout this paper, as a screening-stage approximation rather than as the clinician- and informant-administered CDR itself. Finally, the integration of a deterministic classifier with a conversational interface could, in principle, be explored for other rating-scale instruments—for example, the MoCA, the Geriatric Depression Scale, or the Functional Activities Questionnaire—for the subset of their scoring that is genuinely rule-based; unlike CDR global staging, MoCA and GDS totals are simple sums rather than closed-form categorical rules, so this generalization is a weaker, more speculative analogy than the CDR case and would need to be verified instrument-by-instrument rather than assumed. Prior work combining several of these instruments (MoCA, CDR Global/SB, and the Functional Activities Questionnaire) as joint inputs to an interpretable classifier [22] suggests such extensions are technically feasible, though the deterministic-rule advantage claimed for CDR staging would not automatically transfer to them.

## 5. Conclusions

We have presented a decision tree classifier that reconstructs the global Clinical Dementia Rating from its six sub-domain scores on 13,290 ADNI records, with 99.86% hold-out accuracy under Information Gain and equivalent performance under the Gini Index. The model is embedded within DiAbot, a web-application research prototype in which a large language model administers a CDR-style interview, normalizes the user’s responses to ordinal sub-scores, and submits those sub-scores to the deterministic classifier for staging. At its current stage of validation, DiAbot should be described as a proof-of-concept architecture rather than a deployment-ready or clinically validated tool: clinician-administered comparison, formal inter-rater reliability testing, prospective evaluation with real patients and informants, and a documented data-protection architecture all remain outstanding before any deployment claim can be made. Together, the model and the chatbot constitute an architectural pattern rather than an isolated experimental result and complement the previously published companion study on AD prediction from imaging-derived features in OASIS-2, discussed in Section 4.3.

Several lines of follow-up work are planned. External validation against clinician-administered CDR in a prospective cohort drawn from outside the ADNI is required before any claim of clinical readiness, alongside evaluation of the conversational layer against a much larger, demographically diverse set of real patient interactions, with formal inter-rater reliability analysis against trained clinicians. The system should also be extended to capture longitudinal change flagging clinically significant transitions in CDR-SB from one assessment to the next, which, as discussed in Section 2.1.2, is the form in which the scale is most informative for disease-modifying therapy monitoring, and integrated with the earlier OASIS-2 prediction pipeline discussed in Section 4.3 to yield a single tool that performs both screening and staging against a shared longitudinal patient record. A direct, head-to-head comparison between the trained classifier and a re-implementation of the official rule-based CDR scoring algorithm is also planned, to quantify precisely where and why the two diverge and to demonstrate the added value, if any, of the learned model. Finally, the data-protection architecture required for handling real patient data encryption, authentication, access control, and formal regulatory compliance review will be documented and implemented as part of productionization. Each of these steps brings the system closer to a useful clinical tool.

## Figures and Tables

**Table 1:** Comparison of existing CDR-related web tools with DiAbot.

Platform	CDR Variant	AI/Chatbot	Self-Administered	Result Explanation	History Tracking
Dementia Care Central [16]	CDR-SB	No	Caregiver-mediated	Yes	No
NACC [18]	CDR-SB	No	No (clinician)	No	Research-only
MDCalc [17]	CDR-SB	No	No (clinician)	Yes	No
Brain Health Registry [19]	Embedded items	No	Yes	Limited	Yes
DiAbot (this work)	Full CDR (six domains + global)	Yes (LLM + DT)	Yes	Yes	Yes

**Table 2:** Variables retained for modelling. The CDGLOBAL serves as the target class.

Variable	Description	Type	Range
CDMEMORY	CDR memory domain score	Ordinal	0, 0.5, 1, 2, 3
CDORIENT	CDR orientation domain score	Ordinal	0, 0.5, 1, 2, 3
CDJUDGE	CDR judgement and problem-solving score	Ordinal	0, 0.5, 1, 2, 3
CDCOMMUN	CDR community affairs score	Ordinal	0, 0.5, 1, 2, 3
CDHOME	CDR home and hobbies score	Ordinal	0, 0.5, 1, 2, 3
CDCARE	CDR personal care score	Ordinal	0, 0.5, 1, 2, 3
CDGLOBAL (target)	Global CDR stage	Ordinal (class)	0, 0.5, 1, 2, 3

**Table 3:** Missing-value counts before and after row-wise deletion.

Variable	Missing Before	Missing After
CDMEMORY	79	0
CDORIENT	79	0
CDJUDGE	79	0
CDCOMMUN	80	0
CDHOME	80	0
CDCARE	80	0
CDGLOBAL	84	0

**Table 4:** Aggregate hold-out performance of the two impurity criteria.

Model	Accuracy	Precision (Macro)	Recall (Macro)	F1 (Macro)
Decision Tree, Information Gain	99.86%	99.86%	99.86%	99.86%
Decision Tree, Gini Index	99.79%	99.79%	99.79%	99.79%

**Table 5:** Per-class performance of the Information Gain Decision Tree on the hold-out fold. Support values reflect the stratified test set.

CDR Class	Support (Test)	Precision	Recall	F1
0 (No dementia)	1832	99.95%	99.95%	99.95%
0.5 (Very mild)	1746	99.83%	99.89%	99.86%
1 (Mild)	302	99.67%	99.34%	99.50%
2 (Moderate)	73	98.63%	98.63%	98.63%
3 (Severe)	34	100.00%	100.00%	100.00%

**Table 6:** Ten-fold cross-validation summary. Confidence intervals and a weighted-kappa ordinal agreement statistic are not yet included here (see Section 2.6) and are planned for the next revision.

Model	Mean Accuracy	Std. Dev.	Min. Fold	Max. Fold
DT—Information Gain	99.81%	0.07	99.66%	99.92%
DT—Gini Index	99.74%	0.10	99.57%	99.85%

**Table 7:** Comparison of the proposed model with related studies. Accuracy figures are taken from the original publications and refer to the dataset/target encoding listed.

Study	Dataset	Features	Model	Accuracy	Task Type
Chaves et al. [21]	SPECT	Imaging activations	Apriori AR + SVM	95.87%	AD diagnosis (imaging)
AlShboul et al. [8]	ADNI	CDR + demographics	C4.5 Decision Tree	89.7%	CDR staging (CDR + demographics)
AlShboul et al. [8]	ADNI	CDR + demographics	RIPPER	89.0%	CDR staging (CDR + demographics)
Shoaip et al. [9]	ADNI	ADNI features	JRip + SWRL	92.27%	AD diagnosis (ADNI features)
Shoaip et al. [9]	ADNI	ADNI features	J48 + SWRL	91.47%	AD diagnosis (ADNI features)
Costa et al. [10]	Local cohort	CSF biomarkers	Decision Tree (CART)	74.65%	AD diagnosis (CSF biomarkers)
Naganandhini & Shanmugavadivu [11]	OASIS	Imaging-derived	DTC-HPT	99.01%	AD diagnosis (imaging)
Hashmi [22]	ADNI (1641)	MMSE, CDR Global/SB, MoCA, FAQ, demographics	XGBoost + SHAP (three-class NC/MCI/AD)	94.3%	Three-class diagnosis (NC/MCI/AD)
Yi et al. [23]	ADNI (547)	Neuropsychological + imaging + APOE4	XGBoost + SHAP (three-class NC/MCI/AD)	87.6%	Three-class diagnosis (NC/MCI/AD)
This work (proposed)	ADNI (13,290)	Six CDR sub-domains	Decision Tree (Info Gain)	99.86%	CDR staging (CDR sub-domains)—this work

## Data Availability

The ADNI data used in this study are available from the ADNI Laboratory of Neuro Imaging at https://adni.loni.usc.edu upon registration and approval of a data-use agreement. The pre-processing and modelling code will be made available in the project repository [20] at the time of publication. As of this revision, the repository is not yet public and no URL is available; this statement will be updated with the live address once the repository is released.

## Abbreviations

The following abbreviations are used in this manuscript:

**Abbreviation**	**Definition**
AD	Alzheimer’s disease
ADNI	Alzheimer’s Disease Neuroimaging Initiative
API	Application Programming Interface
CDR	Clinical Dementia Rating
CDR-SB	Clinical Dementia Rating Sum of Boxes
DT	Decision Tree
GDPR	General Data Protection Regulation
HIPAA	Health Insurance Portability and Accountability Act
LLM	Large Language Model
MMSE	Mini-Mental State Examination
MoCA	Montreal Cognitive Assessment
SD	Standard Deviation
SWRL	Semantic Web Rule Language
